# Exploratory Literature Review of the Role of National Public Health Institutes in COVID-19 Response

**DOI:** 10.3201/eid2813.220760

**Published:** 2022-12

**Authors:** Alexandra Zuber, Yesser Sebeh, Dennis Jarvis, Shelly Bratton

**Affiliations:** Ata Health Strategies LLC, Washington, DC, USA (A. Zuber, Y. Sebeh.);; US Centers for Disease Control and Prevention, Atlanta, Georgia, USA (D. Jarvis, S. Bratton)

**Keywords:** COVID-19, 2019 novel coronavirus disease, coronavirus disease, severe acute respiratory syndrome coronavirus 2, SARS-CoV-2, viruses, respiratory infections, zoonoses, public health, prevention and control, national public health institutes

## Abstract

To help explain the diversity of COVID-19 outcomes by country, research teams worldwide are studying national government response efforts. However, these attempts have not focused on a critical national authority that exists in half of the countries in the world: national public health institutes (NPHIs). NPHIs serve as an institutional home for public health systems and expertise and play a leading role in epidemic responses. To characterize the role of NPHIs in the COVID-19 response, we conducted a descriptive literature review that explored the research documented during March 2020–May 2021. We conducted a name-based search of 61 NPHIs in the literature, representing over half of the world’s NPHIs. We identified 33 peer-reviewed and 300 gray articles for inclusion. We describe the most common NPHI-led COVID-19 activities that are documented and identify gaps in the literature. Our findings underscore the value of NPHIs for epidemic control and establish a foundation for primary research.

National public health institutes (NPHIs) are “science-based organizations… that provide leadership and coordination for public health at the national level” (*1*). NPHIs provide an institutional home for many public health functions, which can improve coordination of public health activities; streamline human and financial resources; and improve the generation, sharing, and use of public health data and evidence (*2*–*9*). During public health emergencies, NPHIs can increase countries’ capacity to mount quick, decisive, and coordinated responses (*2*,*3*,*5*,*10*,*11*). An NPHI is often a government agency within a ministry of health but may in some cases represent a parastatal or nongovernmental entity. Approximately half of the countries in the world have an NPHI (n = 94), and they vary in maturity, form, and function (*12*).

Despite their critical role, however, NPHIs have not been a focus of the growing body of research related to characterizing the response to COVID-19 by national governments (*13*–*16*; C.T. Lee et al., unpub. data, https://www.medrxiv.org/content/10.1101/2021.02.02.21251013v1). In 2021, researchers from the World Health Organization and the International Association of National Public Health Institutes (IANPHI) reported that COVID-19 revealed global inequities in public health capacities and established that an “urgent need to examine sources of global knowledge and understand how NPHIs… can be better used, particularly in under-resourced settings” (*17*). To this end, we conducted an exploratory, descriptive literature review to examine 1 question: What clues can the literature give us on the role of NPHIs in the COVID-19 response globally?

## Methods

**Figure 1:**
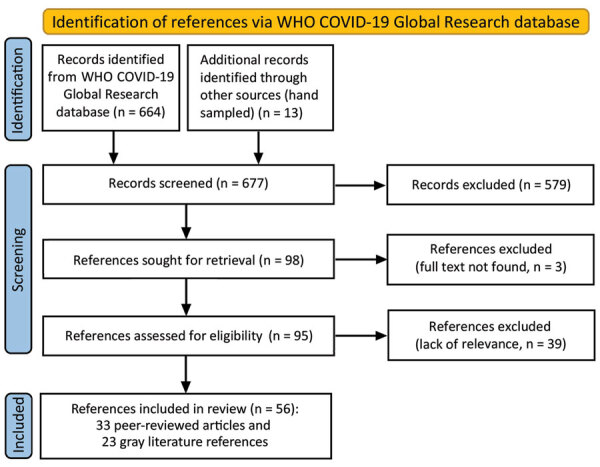
Electronic database search conducted for literature review of the role of national public health institutes in COVID-19 response. Source: (*18*). WHO, World Health Organization**.**

We conducted an electronic database search of articles published in scientific journals (peer-reviewed literature) and a targeted search of documents or reports published outside of academic publishing (gray literature) (Appendix 1). For our electronic search, we selected the World Health Organization COVID-19 Global Research Database on the basis of its comprehensive inclusion of articles from multiple electronic databases and its topical focus on COVID-19 (Figure 1) (*19*). Our search terms (Appendix 2 Table 1) included “national public health institute” as well as the proper names of 61 NPHIs, as listed on the IANPHI website (*12*). We designed a sample frame of these 61 NPHIs by categorizing all 111 IANPHI members by their country’s position on 4 World Bank income levels (i.e., high, upper-middle, lower-middle, and low) and 6 World Bank regions. We then selected 2–3 NPHIs per tier from each of the 6 regions. The NPHIs represented 52 countries because some countries have >1 IANPHI. One researcher conducted the electronic search.

We also searched gray literature for a subsample of 8 NPHIs (selected from the 61 NPHI sample frame). We selected 2 NPHIs from each World Bank income tier, at least 1 per World Bank region. Two researchers searched Google, websites, and social media accounts of the 8 NPHIs. Our Google search terms included the proper name of each of the 8 NPHIs in English, the name in the language of origin, and “COVID-19.” For both searches, we included all studies, reports, new articles, and websites in any language that described activities conducted by NPHIs as part of the COVID-19 response. We used Google Translate for articles not in English (Appendix 2 Table 2).

We imported electronic search articles to NVIVO software (*20*) and gray search articles to an Excel database (Microsoft, https://www.microsoft.com) for qualitative thematic analysis (Appendix 2). We conducted our analysis by following a 3-step, evidence-based strategy (*21*). We used a codebook of deductive and inductive codes and established a coding agreement between reviewer pairs through independent coding and comparison of 2 sample returns. Our conceptual framework was the IANPHI Essential Public Health Functions framework (*22*). This framework describes 11 core public health functions supported by NPHIs, which we used as our exclusive list of deductive codes to categorize NPHI activities in the COVID-19 response (Appendix 2 Table 3).

## Results

### Characteristics of the Literature

From our electronic database search, we screened 667 references by title and abstract and reviewed the full text of 95 articles. A total of 33 peer-reviewed and 23 gray articles met our inclusion criteria. Through our search of gray literature, we identified 277 relevant returns: 75 websites, 62 news articles, 60 social media postings, and 80 guidelines and reports (Appendix 2 Table 4). All articles were published during March 2020–May 2021; 84% were published during June 2020–January 2021.

**Figure 2:**
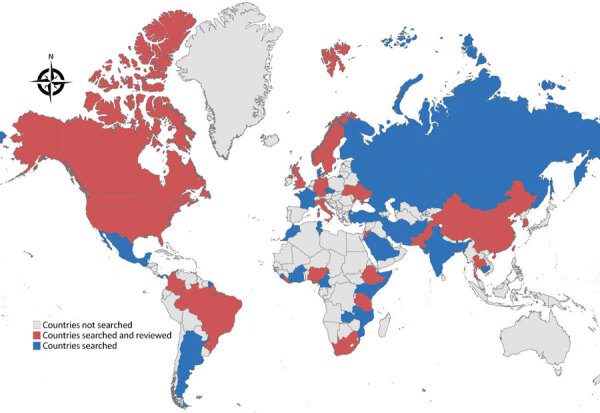
Countries with International Association of National Public Health Institutes members searched and reviewed for literature review of the role of national public health institutes in COVID-19 response.

Articles included in the review described NPHI activities in 20 countries, which represent 39% of the 52 countries searched and 21% of countries globally that have NPHIs (Figure 2; Appendix 2 Table 5). Most articles summarized NPHI activities in a single country (only 3 articles featured NPHI activity in >1 country). The literature from the electronic search was skewed toward 3 countries: Brazil, South Korea, and the United States (representing 33 [59%] of 56 electronic search returns). Returns from the gray literature search of 8 countries represented 236 (71%) of total returns from all searches. As a result, 269 (81%) of the total articles included in the review were focused on 10 countries. The electronic search returned no articles or reports for 34 (65%) of the countries searched.

### NPHI Functions and Activities during the COVID-19 Response

COVID-19 activities among the 20 NPHIs included in this review were reported across all 11 public health functions but most commonly for 5 functions (Appendix 2 Table 3). Because included articles did not document NPHI activities in a consistent fashion across all functions in each country, this summary is an underrepresentation of the full role of each NPHI.

### Public Health Surveillance, Problem Investigation, and Control of Risks and Threats to Public Health

#### Collecting and Sharing Surveillance Data

NPHIs were lead authorities for collecting and analyzing epidemiologic data to project COVID-19 cases, deaths, transmission patterns, and hospitalization rates. To manage COVID-19 data, NPHIs from England and Italy built upon existing integrated disease surveillance systems for infectious disease, including use of sentinel surveillance, vaccine uptake, and household and seroprevalence studies. NPHIs from Canada, Colombia, and Brazil designed and deployed mathematical models to determine scenarios for COVID-19 transmission and to evaluate public health approaches such as quarantine and social distancing. For example, to provide real-time projections of COVID-19 transmission, hospitalizations, and deaths, Brazil used smartphone Global Positioning System data and measured population mobility in combination with COVID-19 deaths, hospital use, and adherence to isolation measures.

#### Setting COVID-19 Case Definitions

For the purposes of disease surveillance, NPHIs set case definitions or standard criteria to classify whether a person has COVID-19. NPHIs in Pakistan, Ethiopia, South Korea, and Jordan established case definitions for screening of passengers at international airports, laboratory and hospital managers of COVID-19 case-patients, and healthcare workers.

#### Managing Laboratory Services

Many NPHIs led laboratory services in the COVID-19 response. For example, the South Korea NPHI partnered with the Korean Society for Laboratory Medicine to develop comprehensive guidelines for laboratory diagnostics for COVID-19, which included selection of persons to test, transport of specimens, diagnostic methods, interpretation of test results, and biosafety. The Pakistan NPHI disseminated standard operating procedures for specimen collection, management, and transport of samples for COVID-19 testing.

Many NPHIs produced the first diagnostic technology for COVID-19 in their countries, including collecting the first samples of COVID-19 and genotyping the virus. The Ethiopia NPHI repurposed existing personnel and infrastructure for malaria, HIV, and other disease research to provide diagnostic capability for COVID-19. The South Korea NPHI leveraged previous efforts to improve coronavirus testing in the wake of the Middle East respiratory syndrome (MERS) epidemic to rapidly establish COVID-19 testing capability as early as December 2019, which enabled extensive early detection of cases. NPHIs from South Korea and Thailand were also involved in genomic sequencing of SARS-CoV-2 virus, which became especially valuable for public health decision-making as new strains emerged.

As COVID-19 cases increased, several NPHIs were at the forefront of COVID-19 case confirmation. The Pakistan NPHI built upon its national public health laboratory and laboratory-based systematic influenza surveillance network to make COVID-19 confirmation testing available by using real-time PCR. Italy NPHI laboratories were opened around the clock to perform confirmation testing; they also provided technical support to other central laboratories for confirmation testing. The Brazil NPHI created COVID-19 Diagnostic Support Units with a testing capacity of 20,000 tests/day.

NPHIs also typically designed and managed the public health laboratory network within each country. The South Korea NPHI ensured that real-time diagnostic capability was established in 18 provincial public health laboratories, and test results became available within 6 hours. The Colombia NPHI first collected all patient samples from 32 departments nationwide for testing in its national reference laboratory; thereafter, it decentralized the process so that ≈172 reference laboratories nationally could support COVID-19 testing. The South Korea NPHI performed quality control of all public and private sector laboratories for in-country COVID-19 diagnostic testing.

#### Screening

NPHIs were engaged in COVID-19 screening of travelers from high-risk countries and of patients, guests, and employees of the hospital system. For example, the US NPHI partnered with the airline industry and other federal authorities to set standards for medical evaluation of passengers before allowing them entry into the country and for mandatory quarantine. Those data were shared with state-level health authorities for follow-up.

#### Testing

NPHIs were lead authorities for COVID-19 testing, which included developing national multisectoral testing plans, overseeing testing facilities, and providing training and technical support to testing facilities across sectors. To improve data matching for results, the England NPHI established procedures for individual self-testing, which included arranging for samples to be sent to the Public Health England national laboratory and linking to the person’s National Health Service identification number. The Liberia NPHI provided COVID-19 testing directly to all incoming air passengers. The Pakistan NPHI monitored subnational testing activities and developed quality indicators for point-of-care testing. To expand COVID-19 testing, it also provided training, technical advice, and support to testing facilities nationwide.

The South Korea NPHI developed a national plan for COVID-19 testing, which included 137 testing facilities across public facilities, public hospitals, and referral laboratories. It also managed an advanced testing network, which included 638 public health centers, a COVID-19 hotline for healthcare providers, and drive-through and walk-through testing centers to enable throughput of patients in ≈10 minutes. Testing strategies in South Korea were also tailored to the level of risk identified by the NPHI, and highly affected regions were targeted for testing by deploying rapid response teams.

#### Quarantine

NPHI support for quarantine activities included helping formulate quarantine policy, providing healthcare service to quarantined populations, and working with government agencies to enforce quarantine. The Liberia NPHI collaborated with county governments and international partners to set up a quarantine facility. The Jordan NPHI provided special medical and healthcare services to quarantined populations. The China and South Korea NPHIs provided data on confirmed cases for local-level police and other authorities to support home-based and facility-based quarantine implementation.

#### Contact Tracing

NPHIs commonly led contact tracing programs. Through the use of technology and wide-ranging multisectoral partnerships, the South Korea NPHI managed a single coordinated contact tracing system that combined smartphone data, credit card transactions, closed-captioned television footage, and more, which enabled public health practitioners to determine a patient’s movement and potential exposures for the past 48 hours. The database also assisted early research on clusters by providing accurate contact mapping. Through international collaboration, the Germany NPHI conducted cross-border contact tracing with other member states in the European Early Warning System and through communication with International Health Regulation national focal points. The China NPHI conducted contact tracing for all confirmed cases in the country identified from its national disease surveillance system.

#### Emergency Operations Centers

Nigeria, the United States, and Ethiopia also led Emergency Operations Centers. In Nigeria, the first confirmed COVID-19 case led to activation of the country’s National Emergency Operations Centre to level 3, and the Nigeria NPHI led this group with the support of Lagos State Health authorities to conduct strict epidemic control measures.

### Public Health Research

NPHIs actively led public health research for COVID-19. NPHIs from Brazil, Colombia, the United Kingdom, South Korea, Norway, Pakistan, Italy, and Canada established networks and platforms for research collaboration. The Norway NPHI established a rapid research review process, which identified evidence needs and conducted evidence reviews in 1–3 days to inform guideline development. All work of this NPHI is published on the Live Map of COVID-19 Evidence, which contained 18,000 publications as of February 2020 (*23*).

NPHIs also conducted research, clinical trials, and published papers related to COVID-19. We found 105 studies with NPHI support, defined as funding (n = 25), data (n = 35), or direct study implementation (n = 13). For example, NPHIs in Colombia, Jordan, and Tanzania conducted seroprevalence studies. NPHIs in Brazil and South Korea conducted clinical trials on treatment, immunization, and mental health effects on healthcare workers as well as epidemiologic studies. NPHIs also made datasets available for other researchers, nationally and internationally.

### Prevention Programs and Health Promotion

NPHIs were further involved in COVID-19 prevention efforts through support for vaccination reporting and risk communication. For example, the US NPHI helped manage 2 vaccine reporting systems to obtain efficacy and safety data on COVID-19 vaccines: the Vaccine Adverse Events Reporting System, which aggregates self-reported adverse vaccine events from patients and clinicians, and the Vaccine Safety DataLink, which gathers hospital data from ≈10 million patients. Both systems enable monitoring of vaccine safety and further studies on rare and severe adverse events. The Colombia NPHI created standard operating procedures for healthcare workers to identify and report vaccine adverse events and register cases with surveillance systems.

NPHIs were involved in risk communication through websites, social media, routine briefings to the public, situational reports, and engagement with communities and multisectoral partners. Health promotion messages and risk communication targeted disproportionately affected populations, such as traditional fishing communities (Brazil), religious congregants (South Korea and Canada), and employees in occupational settings (England). NPHIs’ COVID-19 risk communication activities more commonly focused on a general audience (Italy); restaurants, schools, and nursing homes (Sweden); and other government agencies and clinic settings (United States). In Nigeria, the most popular source of COVID-19 information cited during the pandemic was the NPHI.

NPHIs also worked closely with other sectors and communities to advance their public health messages. The Jordan NPHI started a multisectoral risk-communication campaign on mental health and COVID-19, through partnerships with nongovernmental organizations, academia, public and private media outlets, social media, and religious leaders. The Tanzania NPHI worked with municipalities and local communities to develop a risk communication plan that included relevant media outlets to disseminate culturally appropriate COVID-19 preventive measures. The South Korea NPHI repurposed a 24-hour hotline created for risk communication during the MERS outbreak to support COVID-19 health communication.

### Quality Assurance in Personal and Population-based Healthcare Services

Some NPHIs also supported population access to COVID-19 healthcare services, managed surge capacity, and ensured quality of service delivery. The Brazil NPHI, in partnership with the Ministry of Health, built a rapid assembly hospital on its campus, with 200 beds to treat critically ill COVID-19 patients. The South Korea NPHI established a tiered patient-severity index and supported the repurposing of nonhospital facilities for case-patients with mild illness. Private dormitories and training centers were converted into isolation centers for those with severe illness.

NPHIs also provided national guidance and support for infection prevention and control (IPC) procedures in healthcare and public settings. The Italy NPHI participated in a multisectoral working group that provides guidance on IPC measures against COVID transmission in healthcare facilities and maintained a unit dedicated to the management of IPC initiatives. The South Korea NPHI sterilized and fumigated public places such as public transit settings and theaters.

NPHIs also supported risk assessment in healthcare settings by establishing tools for clinicians and occupational health practitioners. For example, the South Korea NPHI developed standard, mandatory symptom screening of all hospital visitors and staff via a smartphone application. It further reduced hospital-based infections by managing supply and demand of face masks through social networks and smartphone applications.

### Human Resources Development and Training

As part of the COVID-19 response, NPHIs routinely engaged in human resources development, which included training and deploying staff and forming platforms and working groups to coordinate workforce development activities. Ethiopia, Colombia, Liberia, Pakistan, and South Korea NPHIs conducted workshops and training for laboratorians based in universities and hospitals nationwide. NPHIs commonly partnered with other sectors to advance this training. For example, the South Korea NPHI trained private hospitals and laboratories to use the diagnosis kits in partnership with the Korean Society for Laboratory Medicine Practice; the Pakistan NPHI, together with multiple academic partners, provided online training for laboratory technicians.

NPHIs from Canada, Colombia, Italy, Liberia, and Ukraine also built human resource capacity in case identification and management, contact tracing, surveillance, and IPC. The Liberia NPHI leveraged its experience from the Ebola virus disease response to recruit, train, and deploy contact tracers early in the response. The Jordan NPHI and other partners trained ≈400 healthcare workers nationwide on COVID-19 vaccination.

The US NPHI deployed staff to subnational units to assist in the COVID-19 response. It created a dedicated COVID-19 response section to support state, tribal, local, and territorial health departments. The system deployed hundreds of teams to support subnational teams with data collection, epidemiologic investigations, contact tracing, and more.

Two NPHIs managed training platforms and working groups. The Ethiopia NPHI and partners launched the COVID-19 Ethiopia Health Worker Training Platform, a smartphone-based digital learning platform for healthcare workers responsible for COVID-19 diagnosis and treatment. The Italy NPHI supported a multisectoral COVID-19 training working group that designs standardized training methods, conducts needs assessments, evaluates training, and organizes scientific meetings to share knowledge and best practices.

## Discussion

Our literature review revealed that NPHIs played an active role in the COVID-19 response. This role was normative (e.g., setting quarantine policy) and involved implementation (e.g., providing COVID-19 testing). NPHIs rarely acted alone but instead commonly partnered with government authorities at national and subnational levels (including health, education, security, and emergency services); private industry (including private manufacturers, laboratories, and airlines); and civil society (including training institutions, professional associations, and community groups). They also sponsored novel digital health technologies to support contract tracing, quarantine, and population health data analytics.

The engagement of NPHIs in surveillance, public health research, and public health prevention and promotion is consistent with the literature with regard to what are considered core NPHI capabilities (*22*,*24*). However, the active role reported for NPHIs in quality assurance reflects a special role played by NPHIs during an epidemic, in which triaging hospital access and containing hospital-based infections is paramount. Of note, NPHIs routinely leveraged personnel, infrastructure, practices, and policies established in response to previous epidemics (e.g., MERS, HIV, and Ebola) to respond to COVID-19, which illustrates the value of sustained development of epidemic response capability by NPHIs over time.

Limitations of our review included the lack of documentation for 61% of the countries searched and the skew of available articles toward 10 countries, which prevented generalizability of the study findings. It is noteworthy that the highest number of relevant articles was identified by searching NPHI websites and social media, followed by conducting electronic searches by using proper name of the NPHI. Many articles that we screened described the government response to COVID-19 but omitted the role of NPHIs. Few articles offered any comparisons between NPHI activities.

We conclude that there is a gap in the systematic comparison of these institutions with respect to COVID-19, which could elucidate trends, challenges, and best practices in the manner called for by Jakab et al. (*25*). A study by Binder et al., published after our review, contributes to this end (*26*). Those authors conducted a literature review and listening sessions comprising leaders from 10 Africa NPHIs and documented common challenges faced by these NPHIs and innovations. However, they report that their methods did not systematically document NPHI activities with regard to COVID-19, and the article does not document the role of NPHIs outside of Africa (*26*).

To obtain consistent and comprehensive data on the role of NPHIs with regard to COVID-19 globally, we recommend direct data collection through surveys and interviews. Those activities would fill gaps in data by public health function and geography and allow for cross-country comparisons and measuring the degree or intensity of NPHI activities. Survey findings also open up the potential for quantitative analysis of the relationship between NPHI activities and COVID-19 outcomes, such as confirmed cases, mortality rates, and social distancing. Such analyses would benefit from additional information that would enable stratification based on characteristics of NPHIs, such as size, maturity, and funding. Together, this information could build on other analyses that attempt to explain country COVID-19 outcomes (*15*,*16*; C.T. Lee et al. unpub. data, https://www.medrxiv.org/content/10.1101/2021.02.02.21251013v1) and could identify key areas for shoring up public health capacity to improve the response to future epidemics.

Appendix 1 Articles reviewed and cited in literature review of the role of national public health institutes in COVID-19 response**.**

Appendix 2 Additional methods and results for literature review of the role of national public health institutes in COVID-19 response.
