# Supplementary material for: Exploratory Literature Review of the Role of National Public Health Institutes in COVID-19 Response
Source: Emerg Infect Dis. 2022 Dec;28(Suppl 1):S151–8. doi: 10.3201/eid2813.220760 (PMC9745230; doi:10.3201/eid2813.220760)
Supplement: Appendix 1 — Articles reviewed and cited in literature review of the role of national public health institutes in COVID-19 response. [file 22-0760-Techapp-s1.pdf]

Article DOI: https://doi.org/10.3201/eid2813.220760

Exploratory Literature Review of the Role of National Public Health Institutes in COVID-19 Response

Appendix 1

Full list of articles reviewed and cited in the manuscript

1. Ding J, Tuan WJ, Temte JL. Managing close contacts of COVID-19 confirmed cases in metropolitan areas in China. J Public Health Manag Pract. 2020;26:345–8. PubMed https://doi.org/10.1097/PHH.0000000000001189

2. Del Manso M, Andrianou X, Urdiales AM, Vescio MF, Rota MC, Fabiani M, et al.; Gruppo Referenti regionali della Sorveglianza Integrata COVID-19. La sorveglianza integrata Covid-19 in Italia: output e attività correlate [COVID-19 integrated surveillance in Italy: outputs and related activities]. Epidemiol Prev. 2020;44(Suppl 2):70–80. PubMed

3. Radio Diez de Marzo. No es la primera vez que Colombia usa los modelos matemáticos en una epidemia. [This is not the first time that Colombia has used mathematical models in an epidemic] [cited 2021 Jun 2]. https://radiodiezdemarzo.com/no-es-la-primera-vez-que-colombia-usa-los-modelos-matematicos-en-una-epidemia-directora-del-ins

4. National Collaborating Centre for Infectious Diseases (NCCID). PHAC models on COVID-19 [cited 2021 Jun 2]. https://nccid.ca/phac-modelling

5. Barcellos C. Uma nova geografia e o direito à informação e comunicação: a sobrevida em meio à pandemia de Covid-19 [A new geography and the right to information and communication: survival in the midst of the Covid-19 pandemic]. Revista Eletrônica de Comunicação, Informação & Inovação em Saúde. 2020;14 [cited 2021 Jun 2]. https://www.reciis.icict.fiocruz.br/index.php/reciis/article/view/2122

6. Korea Disease Control and Prevention Agency (KDCA). COVID-19 response, Republic of Korea [cited 2021 Jun 2]. http://ncov.mohw.go.kr/en/baroView.do?brdId=11&brdGubun=111&dataGubun=&ncvContSeq=&contSeq=&board_id=&gubun=

7. Ethiopian Public Health Institute (EPHI). EPHI PHEOC COVID-19 [cited 2021 Jun 2]. https://ephi.gov.et/download/ephi-pheoc-covid-19

8. Pakistan Council for Science and Technology (PCST). PCST – Coronavirus Knowledge Centre [cited 2021 Jun 2]. https://www.pcst.org.pk/coronavirus_knowledge_center.php

9. Government of Canada. National case definition: Coronavirus disease (COVID-19) [cited 2021 Jun 2]. https://www.canada.ca/en/public-health/services/diseases/2019-novel-coronavirus-infection/health-professionals/national-case-definition.html

10. Public Health Center (PHC) of Ukraine. COVID-19, case definition [cited 2021 Jun 2]. https://covid19.phc.org.ua/viznachennya-vipadku

11. Lee D, Choi B. Policies and innovations to battle Covid-19—a case study of South Korea. Health Policy Technol. 2020;9:587–97. PubMed https://doi.org/10.1016/j.hlpt.2020.08.010

12. Kim I, Lee J, Lee J, Shin E, Chu C, Lee SK. KCDC risk assessments on the initial phase of the COVID-19 outbreak in Korea. Osong Public Health Res Perspect. 2020;11:67–73. PubMed https://doi.org/10.24171/j.phrp.2020.11.2.02

13. Umair M, Ikram A, Salman M, Khurshid A, Alam M, Badar N, et al. Whole-genome sequencing of SARS-CoV-2 reveals the detection of G614 variant in Pakistan. PLoS One. 2021;16:e0248371. PubMed https://doi.org/10.1371/journal.pone.0248371

14. Tanzania National Institute for Medical Research (NIMR). Advancing health research, enhancing life [cited 2021 Jun 2]. http://www.nimr.or.tz

15. Lim J, Lee J. Current laboratory diagnosis of coronavirus disease 2019. Korean J Intern Med (Korean Assoc Intern Med). 2020;35:741–8. PubMed https://doi.org/10.3904/kjim.2020.257

16. Jung Y, Park GS, Moon JH, Ku K, Beak SH, Lee CS, et al. Comparative analysis of primer-probe sets for RT-qPCR of COVID-19 causative virus (SARS-CoV-2). ACS Infect Dis. 2020;6:2513–23. PubMed https://doi.org/10.1021/acsinfecdis.0c00464

17. Muhammad A, Owais M, Ali N, Khan H. COVID-19 pandemic and precautionary measures in Pakistan. Anaesth Pain Intensive Care. 2020;24:94–100.. https://doi.org/10.35975/apic.v24i1.1231

18. Cohen J. The United States badly bungled coronavirus testing—but things may soon improve. Science Magazine 2020 [cited 2021 Jun 2]. https://pesquisa.bvsalud.org/global-literature-on-novel-coronavirus-2019-ncov/resource/pt/covidwho-2779

19. Badar N, Ikram A, Mirza HA, Ahad A, Alam MM, Arshad Y, et al. Laboratory based surveillance of SARS-CoV-2 in Pakistan. medrxiv.org 2020 Jun 20; https://doi.org/10.1101/2020.06.10.20126847

20. Istituto Superiore di Sanità (ISS). Notiziario, Special Issue: ISS for COVID-19 Vol. 33 [cited 2021 Jun 2]. https://www.iss.it/web/iss-en/notiziario/-/asset_publisher/ATfPbffqTz2N/content/id/5510001

21. Government of Pakistan. Pakistan Preparedness & Response Plan COVID-19. April 24, 2020. [cited 2021 Jun 2]. https://reliefweb.int/report/pakistan/pakistan-preparedness-response-plan-covid-19

22. Abera A, Belay H, Zewude A, Gidey B, Nega D, Dufera B, et al. Establishment of COVID-19 testing laboratory in resource-limited settings: challenges and prospects reported from Ethiopia. Dec 31;13(1). [cited 2021 Jun 2]. https://www.tandfonline.com/doi/abs/10.1080/16549716.2020.1841963

23. Kang YJ. Characteristics of the COVID-19 outbreak in Korea from the mass infection perspective. J Prev Med Public Health. 2020;53:168–70. PubMed https://doi.org/10.3961/jpmph.20.072

24. World Health Organization. Novel coronavirus—Thailand. January 14, 2020. [cited 2021 Jun 2]. https://www.who.int/emergencies/disease-outbreak-news/item/2020-DON234

25. World Health Organization. Novel coronavirus—Republic of Korea. January 14, 2020. [cited 2021 Jun 2]. https://www.who.int/emergencies/disease-outbreak-news/item/2020-DON238

26. Rosa MFF, da Silva EN, Pacheco C, Diógenes MVP, Millett C, Gadelha CAG, et al. Direct from the COVID-19 crisis: research and innovation sparks in Brazil. Health Res Policy Syst. 2021;19:10. PubMed https://doi.org/10.1186/s12961-020-00674-x

27. Hong KH, Lee SW, Kim TS, Huh HJ, Lee J, Kim SY, et al. Guidelines for laboratory diagnosis of coronavirus disease 2019 (COVID-19) in Korea. Ann Lab Med. 2020;40:351–60. PubMed https://doi.org/10.3343/alm.2020.40.5.351

28. Espitia-Almeida F, Pereira-Lentino R, Quintero-Soto J, Gómez-Camargo D. Covid-19 in Cartagena and the Bolívar Department, Colombia. Current status, perspectives and challenges until the arrival of the vaccine. Heliyon. 2021;7:e06336. PubMed https://doi.org/10.1016/j.heliyon.2021.e06336

29. Kang J, Jang YY, Kim J, Han SH, Lee KR, Kim M, et al. South Korea's responses to stop the COVID-19 pandemic. Am J Infect Control. 2020;48:1080–6. PubMed https://doi.org/10.1016/j.ajic.2020.06.003

30. Health Canada and PHAC (@GovCanHealth). Any passengers travelling to Canada who have been in the United Kingdom, South Africa or Brazil in the previous 14 days will be subject to secondary screening and increased scrutiny of quarantine plans. Learn more about quarantine planning here: http://ow.ly/gPnW50DaO5f. Posted January 17, 2021 [cited 2021 Jun 2]. https://twitter.com/GovCanHealth/status/1350909521615577089

31. Issac A, Stephen S, Jacob J, Vr V, Radhakrishnan RV, Krishnan N, et al. The pandemic league of COVID-19: Korea versus the United States, with lessons for the entire world. J Prev Med Public Health. 2020;53:228–32. https://doi.org/10.3961/jpmph.20.166

32. Dollard P, Griffin I, Berro A, Cohen NJ, Singler K, Haber Y, et al.; CDC COVID-19 Port of Entry Team. Risk assessment and management of COVID-19 among travelers arriving at designated U.S. Airports, January 17–September 13, 2020. MMWR Morb Mortal Wkly Rep. 2020;69:1681–5. PubMed https://doi.org/10.15585/mmwr.mm6945a4

33. de Lusignan S, Lopez Bernal J, Byford R, Amirthalingam G, Ferreira F, Akinyemi O, et al. Influenza and respiratory virus surveillance, vaccine uptake, and effectiveness at a time of cocirculating COVID-19: protocol for the English primary care sentinel system for 2020–2021. JMIR Publications 2021;7(2). [cited 2021 Jun 2]. http://publichealth.jmir.org/2021/2/e24341

34. US Embassy in Liberia. Alert: U.S. Embassy Monrovia. August 8, 2020 [cited 2021 Jun 2]. https://lr.usembassy.gov/alert-u-s-embassy-monrovia-august-8-2020

35. Junaidi I. Covid-19 can spread rapidly in winter: NIH - Pakistan Dawn.com. October 8, 2020 [cited 2021 Jun 2].https://www.dawn.com/news/1583789

36. Kang H, Kwon S, Kim E. COVID-19 health system response monitor: Republic of Korea. World Health Organization Regional Office for South-East Asia. 2020. [cited 2021 Jun 2].https://apps.who.int/iris/handle/10665/337371

37. Kim JE, Lee JH, Lee H, Moon SJ, Nam EW. COVID-19 screening center models in South Korea. J Public Health Policy. 2021;42:15–26. PubMed https://doi.org/10.1057/s41271-020-00258-7

38. Partners In Health. PIH Liberia, government partners establish New COVID-19 center. June 2020 [cited 2021 Jun 2]. https://www.pih.org/article/pih-liberia-government-partners-establish-new-covid-19-center

39. World Health Organization. Jordan meets increasing demand for mental health and psychosocial support services during the COVID-19 pandemic through planning and collaboration [cited 2021 Jun 2]. http://www.emro.who.int/ar/mnh/news/jordan-meets-increasing-demand-for-mental-

health-and-psychosocial-support-services-during-the-covid-19-pandemic-through-planning-and-collaboration.html

40. Hong L, Hernandez J. Lessons from abroad: Taiwan's COVID-19 containment model APO.org. May 2020 [cited 2021 Jun 2]. https://apo.org.au/node/303792

41. Markus I, Steffen G, Lachmann R, Marquis A, Schneider T, Tomczyk S, et al. COVID-19: cross-border contact tracing in Germany, February to April 2020. Euro Surveill. 2021;26:1–9. PubMed https://doi.org/10.2807/1560-7917.ES.2021.26.10.2001236

42. Omaka-Amari LN, Aleke CO, Obande-Ogbuinya NE, Ngwakwe PC, Nwankwo O, Afoke EN. Coronavirus (COVID-19) pandemic in Nigeria: preventive and control challenges within the first two months of outbreak. Afr J Reprod Health. 2020;24(s1):87–97. PubMed

43. MedicalBrief. Killer disease stumps Nigeria Centre for Disease Control. February 12, 2020 [cited 2021 Jun 2]. https://www.medicalbrief.co.za/killer-disease-stumps-nigeria-centre-for-disease-control

44. Dirlikov E, Fechter-Leggett E, Thorne SL, Worrell CM, Smith-Grant JC, Chang J, et al.; CDC COVID-19 State, Tribal, Local, and Territorial Response Team. CDC Deployments to state, tribal, local, and territorial health departments for COVID-19 Emergency Public Health Response—United States, January 21–July 25, 2020. MMWR Morb Mortal Wkly Rep. 2020;69:1398–403. PubMed https://doi.org/10.15585/mmwr.mm6939a3

45. Oswaldo Cruz Foundation (Fiocruz). Covid-19 observatory [cited 2021 Jun 2]. https://portal.fiocruz.br/observatorio-covid-19

46. Instituto Nacional de Salud Colombia (INS). @INSColombia realizó taller con entidades territoriales para desarrollar capacidades diagnósticas para #COVID-19 #coronavirus. Posted March 17, 2020 [cited 2021 Jun 2]. https://www.facebook.com/INSColombia/posts/3366291743400291

47. de Souza CTV, de Santana CS, Ferreira P, Nunes JA, Teixeira M LB. da Silveira Gouvêa MIF. Caring in the age of COVID-19: lessons from science and society. Cad Saude Publica. 2020;36:6. https://pubmed.ncbi.nlm.nih.gov/32609168/.

48. Marten R, El-Jardali F, Hafeez A, Hanefeld J, Leung GM, Ghaffar A. Co-producing the covid-19 response in Germany, Hong Kong, Lebanon, and Pakistan. BMJ. 2021;372:n243. PubMed https://doi.org/10.1136/bmj.n243

49. Health Canada and PHAC (@GovCanHealth). The #GoC has mobilized Canada's research and scientific communities in response to #COVID19. Read our research response to covid-19 report

to learn more: http://ow.ly/5Qv650zmQDE. Posted April 24, 2020 [cited 2021 Jun 2]. https://twitter.com/GovCanHealth/status/1253745737944436738

50. Fretheim A, Brurberg KG, Forland F. Rapid reviews for rapid decision-making during the coronavirus disease (COVID-19) pandemic, Norway, 2020. Euro Surveill. 2020;25:1. PubMed https://doi.org/10.2807/1560-7917.ES.2020.25.19.2000687

51. Norwegian Institute of Public Health (NIPH). Map of COVID-19 evidence. June 28, 2021 [cited 2021 Jun 2]. https://www.fhi.no/en/qk/systematic-reviews-hta/map

52. Alcaldía de Santiago de Cali. Instituto Nacional de Salud inicia en Cali estudio de seroprevalencia de covid-19 [National Institute of Health begins in Cali covid-19 seroprevalence study]. November 16, 2020 [cited 2021 Jun 2]. https://www.cali.gov.co/salud/publicaciones/157429/instituto-nacional-de-salud-inicia-en-cali-estudio-de-seroprevalencia-de-covid-19

53. United Nations Jordan. WHO continues supporting Jordan's Ministry of Health response to COVID-19. March 28, 2021 [cited 2021 Jun 2]. https://jordan.un.org/en/123437-who-continues-supporting-jordans-ministry-health-response-covid-19

54. Korea Disease Control and Prevention Agency (KDCA). | : [Clinical trials | Infectious disease: Korea Centers for Disease Control and Prevention] [cited 2021 Jun 2]. https://tinyurl.com/eyp9a8b5

55. Public Health Agency of Canada (PHAC). Preliminary dataset on confirmed cases of COVID-19. August 12, 2022 [cited 2021 Jun 2]. https://www150.statcan.gc.ca/n1/pub/13-26-0003/132600032020001-eng.htm

56. Korea Disease Control and Prevention Agency (KDCA). -19 - [Coronavirus Infectious Disease-19—Press releases] [cited 2021 Jun 2]. http://ncov.mohw.go.kr/tcmBoardList.do?brdId=3&brdGubun=31&dataGubun=&ncvContSeq=&contSeq=&board_id=&gubun

57. Sarpatwari A, Kaltenboeck A, Kesselheim AS. Missed opportunities on emergency remdesivir use. JAMA. 2020;324:331–2. PubMed https://doi.org/10.1001/jama.2020.11932

58. Abiertos D. Casos positivos de COVID-19 en Colombia [cited 2021 Jun 2]. https://www.datos.gov.co/Salud-y-Protecci-n-Social/Casos-positivos-de-COVID-19-en-Colombia/gt2j-8ykr/data

59. Catholic News Agency (CNA). Catholic and Orthodox Churches in Ukraine consider how to distribute Communion. March 13, 2020 [cited 2021 Jun 2].

https://www.catholicnewsagency.com/news/43857/catholic-and-orthodox-churches-in-ukraine-consider-how-to-distribute-communion

60. Public Health Center (PHC) of Ukraine. Сьогодні в. о. генерального директора ЦГЗ Ігор Кузін та начальник відділу управління проектами та міжнародної співпраці Ольга Гвоздецька зустрілися з Митрополит Епіфаній та архієпископом Чернігівським і Ніжинським Євстратієм. Під час зустрічі представники обговорили актуальну епідеміологічну ситуацію в Україні та світі, протидію пандемії COVID-19, зокрема, завдяки вакцинації. Сторони обговорили співпрацю та взаємодію Центру громадського здоров‘я й Православна Церква України. Зокрема, домовилися про продовження популяризації протиепідемічних заходів у релігійних організаціях та дотримання правил у храмах — носіння маски, респіраторна гігієна та гігієна рук, фізична дистанція. Додатково обговорювалося питання вакцинації проти COVID-19 та поширення достовірної інформації серед вірян. Зокрема, для цього Центр і Церква спільно будуть розробляти низку інформаційних матеріалів для прихожан храмів. Posted January 28, 2021 [cited 2021 Jun 2]. https://www.facebook.com/phc.org.ua/posts/2594515074006538?comment_id=2595429240581788

61. The International Association of National Public Health Institutes (IANPHI). IANPHI progress report – 2020. April 22, 2021 [cited 2021 Jun 2]. https://www.ianphi.org/news/2021/2020-progress-report.html

62. Public Health Agency of Canada (PHAC) twitter account (@GovCanHealth) [cited 2021 Jun 2]. https://twitter.com/GovCanHealth

63. Sallam M, Dababseh D, Yaseen A, Al-Haidar A, Ababneh NA, Bakri FG, et al. Conspiracy beliefs are associated with lower knowledge and higher anxiety levels regarding COVID-19 among students at the University of Jordan. Int J Environ Res Public Health. 2020;17:4915.

64. Istituto Superiore di Sanità. COVID-19 integrated surveillance: key national data—EpiCentro March 11, 2020 [cited 2021 Jun 2]. https://www.epicentro.iss.it/en/coronavirus/sars-cov-2-integrated-surveillance-data

65. Instituto Nacional de Salud (INS) Instituto Nacional de Salud twitter account (@INSColombia) [cited 2021 Jun 2]. https://twitter.com/inscolombia

66. National Institute of Health (NIH) Pakistan NIH Pakistan twitter account (@NIH_Pakistan) [cited 2021 Jun 2]. https://twitter.com/NIH_Pakistan

67. National Public Health Institute of Liberia (NPHIL). National Public Health Institute of Liberia twitter account (@NPHIL6) [cited 2021 Jun 2]. https://twitter.com/nphil6

68. Miralles O, Sanchez-Rodriguez D, Marco E, Annweiler C, Baztan A, Betancor É, et al. Unmet needs, health policies, and actions during the COVID-19 pandemic: a report from six European countries. Eur Geriatr Med. 2021;12:193–204. PubMed https://doi.org/10.1007/s41999-020-00415-x

69. Coronado F, Blough S, Bergeron D, Proia K, Sauber-Schatz E, Beltran M, et al. Implementing mitigation strategies in early care and education settings for prevention of SARS-CoV-2 transmission—eight states, September–October 2020. MMWR Morb Mortal Wkly Rep. 2020;69:1868–72. PubMed https://doi.org/10.15585/mmwr.mm6949e3

70. Jeong GH, Lee HJ, Lee J, Lee JY, Lee KH, Han YJ, et al. Effective control of COVID-19 in South Korea: cross-sectional study of epidemiological data. J Med Internet Res. 2020;22:e22103. PubMed https://doi.org/10.2196/22103

71. Marques F. Fiocruz News Agency Oswaldo Cruz Foundation (Fiocruz). November 5, 2020 [cited 2021 Jun 2]. https://agencia.fiocruz.br/alimentacao-na-pandemia-e-o-tema-do-conexao-fiocruz-brasilia

72. Kuhn A. South Korea faces new spike in COVID-19 after months of low infection rates NPR.com. August 17, 2020 [cited 2021 Jun 2]. https://www.npr.org/sections/coronavirus-live-updates/2020/08/17/903157458/south-korea-faces-new-spike-in-covid-19-after-months-of-low-infection-rates

73. Public Health Center (PHC) of Ukraine. Ukraine has submitted the first part of the application for a vaccine against COVID-19 Center for public health. December 8, 2020 [cited 2021 Jun 2]. https://phc.org.ua/news/ukraina-podala-pershu-chastinu-zayavki-na-otrimannya-vakcini-vid-covid-19

74. Infobae. Instituto Nacional de Salud alerta sobre la “tormenta perfecta” que sería el covid-19 durante la Semana Santa en Colombia—Infobae. March 26, 2021 [cited 2021 Jun 2]. https://www.infobae.com/america/colombia/2021/03/26/instituto-nacional-de-salud-alerta-sobre-la-tormenta-perfecta-que-seria-el-covid-19-durante-la-semana-santa-en-colombia

75. Paton N. Managing Covid-19 “clusters” set to be a key challenge for occupational health—Personnel Today. Occupational Health & Wellbeing Plus. October 2, 2020 [cited 2021 Jun 2].

https://www.personneltoday.com/hr/managing-covid-19-clusters-set-to-be-a-key-challenge-for-occupational-health

76. Tagliacozzo S, Albrecht F, Ganapati NE. International perspectives on COVID-19 communication ecologies: public health agencies' online communication in Italy, Sweden, and the United States. Am Behav Sci. 2021;65:934–55. https://doi.org/10.1177/0002764221992832

77. Apuke OD, Bahiyah O. How do Nigerian newspapers report COVID-19 pandemic? The implication for awareness and prevention. Health Educ Res. 2020;35:471–80 [cited 2021 Jun 2]. https://academic.oup.com/her/article-abstract/35/5/471/5935539

78. Mboya IB, Ngocho JS, Mgongo M, Samu LP, Pyuza JJ, Amour C, et al. Community engagement in COVID-19 prevention: experiences from Kilimanjaro region, northern Tanzania. Pan Afr Med J. 2020;35(Suppl 2):146. PubMed https://doi.org/10.11604/pamj.supp.2020.35.2.24473

79. Song R, Kim HS, Yoo SJ, Lee K, Park JH, Jang JH, et al. COVID-19 in nursing facilities: experience in Republic of Korea. Osong Public Health Res Perspect. 2020;11:164–9. PubMed https://doi.org/10.24171/j.phrp.2020.11.4.04

80. Oswaldo Cruz Foundation (Fiocruz). UNITED AGAINST COVID-19 [cited 2021 Jun 2]. https://unidos.fiocruz.br/index-en.html

81. Kang YJ. Lessons learned from cases of COVID-19 infection in South Korea. Disaster Med Public Health Prep. 2020;14:818–25. PubMed https://doi.org/10.1017/dmp.2020.141

82. United Nations Population Fund. Learning from Ebola experience, Liberia deploys contact tracers to break COVID-19 transmission. Africa Renewal. April 24, 2020 [cited 2021 Jun 2]. https://www.un.org/africarenewal/news/learning-ebola-experience-liberia-deploys-contact-tracers-break-covid-19-transmission

83. Last Mile Health. COVID-19 response [cited 2021 Jun 2]. https://lastmilehealth.org/what-we-do/covid-19-response

84. Devex. National Public Health Institute of Liberia (NPHIL) [cited 2021 Jun 2]. https://www.devex.com/organizations/national-public-health-institute-of-liberia-nphil-132858

85. Hur JY, Kim KW. Crisis learning and flattening the curve: South Korea's rapid and massive diagnosis of the COVID-19 infection. Am Rev Public Adm. 2020;50:606–13. https://doi.org/10.1177/0275074020941733

86. Sung H, Yoo CK, Han MG, Lee SW, Lee H, Chun S, et al. Preparedness and rapid implementation of external quality assessment helped quickly increase COVID-19 testing capacity in the Republic of Korea. Clin Chem. 2020;66:979–81. PubMed https://doi.org/10.1093/clinchem/hvaa097

87. Bhutta ZA, Sultan F, Ikram A, Haider A, Hafeez A, Islam M. Balancing science and public policy in Pakistan's COVID-19 response. East Mediterr Health J. 2021;27:798–805. PubMed https://doi.org/10.26719/emhj.21.016

88. The Aga Khan University. News [cited 2021 Jun 2]. https://www.aku.edu/news/Pages/home.aspx

89. World Health Organization. Nurses and midwives are critical to teaching hand hygiene in Pakistan. May 4, 2020 [cited 2021 Jun 2]. https://www.who.int/news-room/feature-stories/detail/nurses-and-midwives-are-critical-to-teaching-hand-hygiene-in-pakistan

90. The Public Health Agency of Canada (PHAC). COVID-19 - Free contact tracing training opportunities. 2020 [cited 2021 Jun 2]. http://www.afn.ca/wp-content/uploads/2020/10/CoronaVirus_Free__Contact_Tracing_ENG.pdf

91. Gerrard A. A coordinated public-private sector response in Liberia to COVID-19 Health Policy Plus [cited 2021 Jun 2]. http://www.healthpolicyplus.com/liberiaHFL.cfm

92. Petrini C, D'Aprile C, Floridia G, Gainotti S, Riva L, Tamiozzo S. Protection of individual and collective health: ethical-legal issues and opportunities for public health after COVID-19 [cited 2021 Jun 2]. https://pesquisa.bvsalud.org/global-literature-on-novel-coronavirus-2019-ncov/resource/pt/covidwho-1139101

93. Oswaldo Cruz Foundation (Fiocruz). Recomendações para o Método Canguru Durante a Pandemia de COVID-19 Portal of Good Practices in Women's, Children's and Adolescents' Health. March 2020 [cited 2021 Jun 2]. https://portaldeboaspraticas.iff.fiocruz.br/biblioteca/recomendacoes-para-o-metodo-canguru-durante-a-pandemia-de-covid-19

94. Kim EY, Kim EK, Lee M, Park HK. COVID-19 public health measures during national assembly elections of the Republic of Korea. Osong Public Health Res Perspect. 2020;11:158–63. PubMed https://doi.org/10.24171/j.phrp.2020.11.4.03

95. Song R, Choi YS, Ko JY. Operating a national hotline in Korea during the COVID-19 pandemic. Osong Public Health Res Perspect. 2020;11:380–2. PubMed https://doi.org/10.24171/j.phrp.2020.11.6.06

96. Andersson S, Aylott N. Sweden and coronavirus: unexceptional exceptionalism. Soc Sci. 2020;9:232. https://doi.org/10.3390/socsci9120232

97. Onalu CE, Chukwu NE, Okoye UO. COVID-19 response and social work education in Nigeria: Matters arising. Soc Work Educ. 2020;39:1037–47. https://doi.org/10.1080/02615479.2020.1825663

98. Bledsoe M, Captanian A. research ASJ of safety, 2021 undefined. Special report from the CDC: strengthening social connections to prevent suicide and adverse childhood experiences (ACEs): actions and opportunities. J Safety Res. 2021;77:328–33. PubMed https://doi.org/10.1016/j.jsr.2021.03.014

99. Kesselheim AS, Darrow JJ, Kulldorff M, Brown BL, Mitra-Majumdar M, Lee CC, et al. An overview of vaccine development, approval, and regulation, with implications For COVID-19. Health Aff (Millwood). 2021;40:25–32. **PMID: 33211535**

100. Zhang Y, Shi L, Chen H, Wang X, Sun G. Policy disparities in response to the first wave of COVID-19 between China and Germany. Int J Equity Health. 2021;20:86. PubMed https://doi.org/10.1186/s12939-021-01424-3
