# Supplementary material for: Exploratory Literature Review of the Role of National Public Health Institutes in COVID-19 Response
Source: Emerg Infect Dis. 2022 Dec;28(Suppl 1):S151–8. doi: 10.3201/eid2813.220760 (PMC9745230; doi:10.3201/eid2813.220760)
Supplement: Appendix 2 — Additional methods and results for literature review of the role of national public health institutes in COVID-19 response. [file 22-0760-Techapp-s2.pdf]

Article DOI: https://doi.org/10.3201/eid2813.220760

Exploratory Literature Review of the Role of National Public Health Institutes in COVID-19 Response

Appendix

Methods Summary

Data collection

Our descriptive literature review aimed to characterize the role of NPHIs in COVID-19 and identify any gaps in the literature on this subject. The review was not a systematic review, but did follow many PRISMA checklist practices. Our research team comprised three researchers. Our methods included an electronic database search of peer-reviewed articles (Appendix Table 4) and gray articles, as well as search for gray reports. We conducted our electronic database search using the WHO COVID-19 Global Research Database. We selected the WHO COVID-19 Global Research Database due to its comprehensive inclusion of articles from multiple electronic databases, and its focus on articles pertaining to COVID-19.

In the WHO COVID-19 Global Research Database, we conducted several consecutive searches. The first search used the terms "national public health institute," with a filter of "title, abstract, or subject". No other filters were used, such as to narrow by language, database, or period of publication. We then established a sample frame of NPHIs to represent a majority of NPHIs in the world. We categorized all IANPHI members (n=111) by their country's position on four World Bank income levels (i.e., high, upper middle, lower middle, and low income) and six World Bank regions. We then purposely selected two to three NPHIs per tier from each of the six regions, which resulted in 61 NPHIs selected. We selected NPHIs in the pattern we felt balanced geography and income levels most equally. The NPHIs represented 52 countries because some countries have more than one IANPHI member.

We then conducted 61 consecutive discrete searches each using the proper name of the NPHIs, in English and in the official language of its host country, as listed on the IANPHI

website. The same filter of "title, abstract, or subject" was used for each of these searches, and no others. The period for these searches was March 19-23, 2021 (search term "NPHI") and May 3-7, 2022 (proper name searches). One researcher conducted the name-based search and suggested articles for inclusion in three categories: yes, no, or maybe. The senior author then reviewed all categories and the two decided together on a call the articles for inclusion and exclusion.

Our inclusion criteria (summarized in Appendix Table 2) was that the article had the search term in the title or abstract of the article, described the role of the NPHI or NPHIs in responding to COVID-19, had as a study context one of the 61 countries in our sample frame, comprised quantitative or qualitative studies, technical reports, assessments or evaluations, new reports, social media posts, or government websites; and are written in any language. Google Translate was used for languages other than English, French, Arabic, and Portuguese. Our exclusion criteria was publications that did not provide sufficient information on the NPHI's role, publications that summarized studies that did not include the NPHI as a funder, implementer, or provider of data, and full text documents that were not accessible.

We recognized that because of the early advent of COVID-19, NPHI activities may not yet be documented in the peer-reviewed literature. Therefore, we constructed a gray literature search strategy, to identify reports, assessments, news articles and more that would summarize NPHI activities. For feasibility reasons, we narrowed our gray search sample frame to eight NPHIs (selected from the 61 NPHI sample frame). We selected two NPHIs from each World Bank income tier, with at least one per World Bank region. We aimed to select the list of countries that best represented geographic and income diversity. For example, if we selected a country with 'high -middle' income in one region, we would attempt to select a country with 'low-middle' income in another, so that income characteristics were evenly distributed. Two researchers conducted the gray search, and independently determined inclusion or exclusion of articles based on our common criteria.

Once the study frame was established, we searched Google, as well as the official government websites and social media accounts, of the eight NPHIs. Our Google search terms included the proper name of each of the eight NPHIs in English, as well as in the language of origin, AND "COVID-19." We included all studies, reports, new articles, and web pages in any

language that described activities conducted by NPHIs as part of the COVID-19 response. We used Google Translate for articles not in English. The same inclusion and exclusion criteria that was used for the electronic search was used for the gray search. The dates for this gray literature search was June 1, 2021- June 8, 2021.

Data Analysis

Articles form the electronic search were imported to NVIVO software (*19*) for qualitative analysis. We utilized a 3-step, evidence-based strategy described by Forman and Damschroeder, 2008. The first step (immersion) included review of the article in its entirety, at times taking any notes or observations in a linked memo in NVIVO. In the second step – "deduction"- the authors conducted "preliminary coding" of each article (and memo where it existed) using the deductive parent codes. Once all articles had been coded by deductive parent codes, the authors then saved a PDF file of each parent "node" report, and conducted secondary coding of each of these reports. The authors identified inductive sub-codes in this process, and coded each report accordingly to those nodes. In the third and final stage – interpretation- the authors opened each of the sub-code reports and discussed findings, developed conclusions, and identified country examples to highlight in the narrative.

For the gray returns, our research pair preferred the use of Microsoft Excel for data management. Given the volume of returns, it was more efficient to cut and paste coded passages to Excel than attempting to import webpages and other online documents to NVIVO. Coding was conducted by two researchers, who were responsible for analysis of four countries each. They reviewed each return in full, then reviewed a second time, cutting and pasting coded passages in the Excel fields under parent and secondary code columns. Passages were further organized by source (with link), country, Google results page number, and date of publication where available. Secondary codes were those identified inductively from the analysis of the electronic search returns. Using column filters, the team was able to quantify the number of returns that cited NPHI activities in each of the parent and secondary codes, and these numbers were consolidated with the numbers determined from NVIVO to complete Table 3.

We used a codebook of deductive and inductive codes. Our conceptual framework was the IANPHI Essential Public Health Functions framework (*23*). This framework describes 11 "core" public health functions supported by NPHIs, which we used as our deductive codes to

categorize NPHI activities in the COVID-19 response. We relied on the definitions provided by IANPHI for these functions to support inter-rater reliability. No other deductive codes were used. We established coding agreement by having each review pair, of which there were two, independently code two returns, compare coding passages and discuss and resolve differences in interpretation.

Because this was a descriptive and not a systematic review or meta-analysis, we did not feel it was appropriate to evaluate the quality of the articles. The articles we identified were of sufficient diversity in type and content, rendering quality scoring impossible. However, each of three reviewers was asked to assess the "relevance" of the article findings to the study topic for each of the peer-reviewed articles. This data, along with a summary of key related findings of each article, is included as a table appended to the manuscript.

Data visualization

The authors took select steps to visualize the data generated. First, one researcher established a table of the countries that were searched and the countries that had articles included in the review, and converted this data into a color-coded map. The authors also used NVIVO data to quantify the number of articles that cited at least one activity in a particular parent code, and assembled this tabulation in a table appended to the manuscript. This table summarizes the total number of articles that cite NPHI activities, by each of 11 essential public health functions.

Appendix Table 1. Search terms

Method	Database	Search terms	Dates
Electronic database search	WHO COVID-19	"national public health institute"	May 26, 2021
	WHO COVID-19	Proper name of NPHIs in 61 countries in English and then in the language of origin (Table)	May 26[h] and August 1, 2021
Search of the gray literature	Google	For 8 select NPHIs, applied proper name of the NPHI in English OR the language of origin AND COVID-19	June 1st, 2021 to June 8th, 2021
	NPHI websites, Twitter & Facebook	For 8 select NPHIs, conducted hand-search for articles, reports, or news stories describing activities of one of eight select NPHIs the COVID-19 response. Facebook posts from January 2020 to March 2021 were reviewed.	June 1st, 2021 to June 8th, 2021

Appendix Table 2. Inclusion and exclusion criteria for screened articles

Type of criteria	Specific criteria
Inclusion criteria	
	Have the search term in the title or abstract of the article.<br>Describe the role of the NPHI or NPHIs in responding to COVID-19.<br>Study context is one of the 61 countries of focus for our review.<br>Comprised quantitative or qualitative studies, technical reports, assessments or evaluations, news reports, social media posts, or government websites.<br>Are written in any language; Google Translate was used for languages other than English, French, Arabic, and Portuguese.
Exclusion criteria:	
	Publications that did not provide sufficient information on the NPHI's role<br>Publications that summarized studies that did not include the NPHI as a funder, implementer, or provider of data.<br>Full text documents are not accessible

Appendix Table 3. Articles included in the review that document an NPHI's role in the COVID-19 response by IANPHI Core Public Health Function

IANPHI Public Health Functions	No. of returns from the electronic database search	No. of returns from the search of websites and social media
1. Evaluation and analysis of health status	4	3
2. Public health surveillance, problem investigation, and control of risks and threats to public health	30	53
3. Prevention programs and health promotion	24	32
4. Social participation in health	7	13
5. Planning and management	7	4
6. Regulation and enforcement	5	4
7. Evaluation and promotion of coverage and access to health services	3	5
8. Human resource development and training	6	0
9. Quality assurance in personal and population -based health services	11	6
10. Public health research	10	7
11. Reduction of the impact of emergencies and disasters on health	5	10

*A single article may refer to more than one function, so the total of each column is over the total number of articles reviewed.

Appendix Table 4. Peer-reviewed articles from the literature review of the role of NPHIs in the COVID-19 response

			Quality assessment	
Reference	Country	Relevant findings	Type of reference	Relevance
(De Lusignan et al., 2021) (*1*)		Throughout the paper, the authors mention and describe the linkage with Public Health England (PHE). PHE is described as conducting surveillance and research activities. Within the surveillance function, PHE has the lab capacity to collect self-administered COVID-19 swabs and analyze them to provide results. PHE is also a member of I-MOVE (Influenza–Monitoring Vaccine Effectiveness in Europe) consortium for monitoring influenza vaccine effectiveness in Europe.	Peer reviewed article	Medium
(Del Manso et al., 2020) (*2*)	Italy	ISS activities included defining and updating case definitions, data sharing, setting up technology to enhance the data shared, quality and completeness of the data, and integrating COVID-19 reporting to the IDSR. The paper mainly discusses these IANPHI core functions: evaluation and analysis of health status, public health surveillance, problem investigation, and control of risks and threats to public health, social participation in health, and reduction of the impact of emergencies and disasters on health.	Peer reviewed article	High
(Markus et al., 2021) (*3*)	Germany	RKI managed the data sharing between the public authority in Germany and abroad. Cross border contact tracing at the national level is operated by the Robert Koch Institute (RKI), the federal public	Peer reviewed article	Medium

Reference	Country	Relevant findings	Quality assessment: Type of reference	Quality assessment: Relevance
		health institute in Germany. Besides that, the paper has no additional examples besides a flow chart of information in the context of contact tracing in Germany.		
(Petrini et al., 2020) (*4*)	Italy	The report contains guidance and ethical considerations after the COVID-19 pandemic geared towards understanding the "Bioethical aspects" that the general practitioner faces when treating patients, family, and the community, Family nurses, telemedicine, the reform of the electronic health records, delayed care due to the pandemic and lockdowns, the right to health, protection of vulnerable populations, palliative care, and contact tracing.	Report	Low
(Espitia-Almeida et al., 2021) (*5*)	Colombia	The INS was the regulation body that gave approvals to approve COVID-19 samples. The samples in the study were transported from provider institutions (IPS) then to departmental public health labs, then UNIMOL labs (process), then results were updated in a national registry platform. The role of the INS was to process all the results.	Peer reviewed article	High
(Abera et al., 2020) (*6*)	Ethiopia	The EPHI was able to identify a suitable space, mobilize the necessary resources, and train staff. A steering committee was formed by voluntary team members from the Malaria and NTDs research team to lead the laboratory establishment. Some of the lessons learned include: 1) Repurpose existing laboratory spaces using national and WHO guidelines 2) Mobilize underutilized resources such as equipment and human resources for COVID-19 laboratory setup. (particularly equipment available for health research and diagnostics); 3) Collaborate with local and international health experts and equipment manufacturers and agents to solve laboratory issues 4) Ensure that space and equipment identified for COVID-19 testing is sterile and contamination free from the outset. The paper also suggests that university can follow a similar process to establish COVID-19 rapid testing laboratories.	Peer reviewed article	High
(Fretheim et al., 2020) (*7*)	Norway	All reviews are published with explicit messages about the risk of overlooking key evidence or making misguided judgements by using such rapid processes. The NIPH established a rapid review team with a 1 to 3 days turnaround time.	Commentary article	High
(Fiocruz, 2020) (*8*)	Brazil	In Brazil, FIOCRUZ worked with a group of national consultants to produce a report containing recommendations when caring for newborns in the context of COVID-19. The NPHI was involved with research and compiled existing recommendations. Additionally, FOCRUZ set up case definitions.	Report	Low
(K. H. Hong et al., 2020) (*9*)	S. Korea	Korean Society for Laboratory Medicine and the Korea Centers for Disease Prevention and Control proposed guidelines for diagnosing COVID-19 in clinical laboratories in Korea. These guidelines are based on other related domestic and international guidelines, as well as expert opinions and include the selection of test subjects, selection of specimens, diagnostic methods, interpretation of test results, and biosafety.	Peer reviewed article	High
(Hur & Kim, 2020) (*10*)	S. Korea	Crisis learning enabled South Korea to flatten the COVID-19 infection curve—specifically, by applying lessons from both past epidemics and the current outbreak. After the country suffered from 2015 Middle East respiratory syndrome coronavirus (MERS) outbreak, the Korea Centers for Disease Control & Prevention (KCDC), the national disease control agency, improved its surveillance system by establishing a 24-hr Emergency Operation Center (EOC) to collect information about real-time domestic and international infectious diseases. Moreover, the KCDC adopted measures such as emergency use authorization (EUA) to speed up the development and the supply of diagnosis equipment, which enabled qualified private health providers to diagnose cases of the viral infection.	Peer reviewed article	High
(Issac et al., 2020) (*11*)	S. Korea	Korea KDCA developed the mobile application “Corona-100m”, which alerted people who came within 100 meters from places where confirmed cases had been. Additionally, they conducted contact tracing of cases and tested asymptomatic people regardless of their contacts. The transparency of the agency was also mentioned as crucial to the response (media briefings).	Commentary article	High

Reference	Country	Relevant findings	Quality assessment<br>Type of reference	 Relevance
(Jeong et al., 2020b) (*12*)	S. Korea	In South Korea, the CDSCHQ is operated by the Ministry of Health and Welfare and Ministry of Public Administration and Security, to support KCDC with its disease control efforts and to provide the necessary assistance in matters requiring coordination between the central government and local municipal governments. A confirmed patient is reported as soon as diagnosed to the provincial government and KCDC immediately.	Peer reviewed article	High
(Y. J. Kang, 2020) (*13*)	S. Korea	The KCDC was able to approve the use of a testing kit and rapidly deploy it to around 50 testing facilities. Other key steps were tracking cases, finding exposed individuals, coordinating case assignments with health care facilities, and selective clinic screenings for visitors' entering hospitals with mandatory mask wearing. Korea KDCA was involved in: updating case definitions, expanding diagnosis testing, tracking exposed cases and isolation of confirmed cases, and treatment, Public messaging	Peer reviewed article	High
(E. Y. Kim et al., 2020) (*14*)	S. Korea	The KCDC personnel coordinated with the government departments related to the election such as the Ministry of the Interior and Safety, local governments, and the National Election Commission. The KCDC established guidelines for COVID-19 patients and individuals isolating at home during the general elections for the 21st National Assembly.	Peer reviewed article	High
(I. Kim et al., 2020) (*15*)	S. Korea	The KDCA established risk assessment criteria that aimed to provide information to enable evidence-based strategic response planning and relevant response measures for KCDC and the Ministry of Health and Welfare. It provided the risk assessment for COVID-19 at given dates, including the details on travel-associated imported cases, clusters outside Korea, risk for healthcare system capacity, options for preparedness and response, risk communication, social distancing, contact tracing and enhanced surveillance.	Peer reviewed article	High
(D. Lee & Choi, 2020) (*16*)	S. Korea	The KCDC jointly with medical professionals developed a series of innovations such as 1) full contact tracing and rapid testing with a 12 h turnaround and 10 min movement tracking systems, 2) transparent disclosure of all contract tracing data to the public through a central database, 3) drive through and walk- Through testing methods, and 4) a 4-tier patient severity index and community treatment isolation centers. Korea moved from the 4th in the world for total confirmed cases in March down to 76th in August.	Peer reviewed article	High
(Song et al., 2020) (*17*)	S. Korea	The KCDC operated and managed a national hotline, first created during a previous epidemic. The KCDC also engaged in health promotion activities with the participation of EIS staff. In its activities, the KCDC engaged with government ministries and financial investment firms.	Journal article	Low
(Rosa et al., 2021) (*18*)	Brazil	In Brazil, Fiocruz engaged in research related activities in collaboration with universities in the country.	Commentary article	Low
(Song et al., 2020) (*19*)	S. Korea	In Korea, the KCDC's preventive measures guidance is to test all the people who have been in contact with a confirmed COVID-19 case.	Peer reviewed article	High
(de Souza et al., 2020b) (*20*)	Brazil	In Brazil, Fiocruz engaged with community leaders to counter fake information on COVID-19. They used WhatsApp in their methodology.	Journal article	Medium
(Andersson & Aylott, 2020a) (*21*)	Sweden	The Swedish Institute, a public agency that, according to its website, "promotes interest and trust in Sweden around the world". The Swedish institute participated in the planning and management efforts in the country, assessed the risk of COVID-19 infections, and produced guidelines and recommendations. The Agency also recommended social distancing measures and other preventions measures.	Peer reviewed article	Medium
(Onalu et al., 2020) (*22*)	Nigeria	The Nigeria Centre for Disease Control [NCDC] was at the forefront of providing information about the virus and required preventive measures for the public.	Peer reviewed article	Low
(Apuke & Omar, 2020) (*23*)	Nigeria	This study examined media coverage of COVID19 in Nigeria with attention to the frequency and depth of coverage, story format, news sources, media tone and themes. The highest source cited was the NCDC, followed by other government officials, health sector, medical experts (e.g., virologists) and WHO and United Nations. They announce new infectious disease, death rates and any other related cases including COVID-19 infections.	Peer reviewed article	Medium

Reference	Country	Relevant findings	Quality assessment: Type of reference	Quality assessment: Relevance
(Bledsoe et al., 2021) (*24*)	United States	The US CDC provided guidance for suicide prevention in the context of COVID-19 pandemic (due to self- isolation).	Peer reviewed article	Low
(Coronado, 2020) (*25*)	United States	MMWR report on the implementation of mitigation strategies in education settings. The US CDC played a role of setting guidelines to be implemented. These guidelines were linked to federal funding.	Peer reviewed article	Medium
(Ding et al., 2020) (*26*)	China	The Chinese Center for Disease Control and Prevention (CCDC) has required that all COVID-19 confirmed cases be recorded and documented in a national notifiable disease surveillance system (NDSS). The CCDC also performed surveillance and contact tracing activities. Additionally, the CCDC adopted a community-based approach in contact tracing involving all stakeholders including local public health departments, public safety authorities, neighborhood councils, and community health centers.	Peer reviewed article	Medium
(Dirlikov et al., 2020) (*27*)	United States	MMWR report on the deployment of US CDC staff to subnational level health authorities. After activating the EOC, the US CDC established a dedicated COVID-19 response section to support state, tribal, local, and territorial health departments. The US CDC provided the following assistance: epidemiologic support, infection prevention and control in health care settings, health communications, community mitigation, and occupational safety and health	Peer reviewed article	High
(Dollard et al., 2020) (*28*)	United States	MMWR report on the risk assessment and management of COVID-19 at US airports. The US CDC, in collaboration with the department of homeland security, instituted a screening program for air travelers into the United States. Th US CDC also shared the data with states' health departments to better control the spread of the virus via CDC's Epidemic Information Exchange (Epi-X).	Peer reviewed article	High
(Kesselheim et al., 2021) (*29*)	United States	The US CDC, alongside the FDA takes part in the post approval surveillance and safety system for vaccines. The surveillance is based on the following systems: CDC and FDA Vaccine Adverse Event Reporting System (VAERS), the CDC Vaccine Safety Datalink, and the CDC Clinical Immunization Safety Assessment (CISA) Project.	Peer reviewed article	Medium
(Miralles et al., 2021) (*30*)	Belgium, France, Italy, Poland, Spain, and United Kingdom	The article assesses the impact of policies to decrease the overall impact of the COVID-19 pandemic in Europe on the older population. From the 6 European countries: Belgium, France, Italy, Poland, Spain, and United Kingdom, only one NPHI example was mentioned. Participation in providing information to the public through the use of websites and social media outlets and health promotion activities were portrayed.	Peer reviewed article	Low
(Omaka-Amari et al., 2020) (*31*)	Nigeria	The Nigeria CDC led the activation of the country's EOC, contact tracing, testing, isolation, and providing information to the public about the risks of the spread of the virus.	Peer reviewed article	Low
(Tagliacozzo et al., 2021) (*32*)	Italy, Sweden, United States	This study examines the online communication of national public health agencies during the COVID-19 pandemic in Italy, Sweden, and the United States. NPHIs from these countries took part in intergovernmental efforts to provide information to the public through their social media outlets and collaborated with National NGOs. These NPHIs mainly targeted the general public, businesses, nursing homes, etc.	Peer reviewed article	Medium
(Zhang et al., 2021) (*33*)	China, Germany	This article summarized policy disparities in response to the first wave of COVID-19 between China and Germany. German Federal Center for Disease Control (Robert Koch Institute) assessed the situation of COVID-19 in Germany. The Ministry of health and defense used those assessments to establish a federal-level epidemic response headquarters and developed a series of prevention and control measures.	Peer reviewed article	Low

* Quality assessment was not deemed appropriate for this descriptive review, as our aim was to describe the role of NPHIs in the COVID-19 response using a diverse range of article types and not to compare or contrast the impact of interventions. However, we characterize the literature by documenting their study type, country of focus, their relevant findings, and the degree (high, medium to low) to which they described an NPHIs role in COVID-19 response (i.e. were relevant).

Appendix Table 5. Countries (n = 52) and IANPHI members (n = 61) searched, and the articles identified by country in the electronic database search and search of gray literature

Count	Country	Name of NPHI *(Based on IANPHI member list)*	Acronym	No. of returns included in the electronic database search	No. of articles included in the search of websites, and social media	NPHI Websites searched
1.	Afghanistan	Afghan National Public Health Institute	NPHI	0	*	*
2.	Argentina	Administración Nacional de Laboratorios e Institutos de Salud	ANLIS	0	*	*
3.	Bangladesh	Institute of Epidemiology Disease Control & Research *(IEDCR)*	IEDCR	0	*	*
4.	Brazil	Fundação Oswaldo Cruz	FIOCRUZ	16	*	*
5.	Burkina Faso	Institut National de Sante Publique	INSP	0	*	*
6.	Cambodia	National Institute of Public Health	NIPH	0	*	*
7.	Cameroon	Direction de la Lutte Contre la Maladie, les Epidemies, et les Pandemies	DLM	0	*	*
8.	Canada	Institut National De Santé Publique Du Quebec	INSP	0	41	https://www.canada.ca/en/public*health.html
9.	China	Chinese Center for Disease Control and Prevention	C. CDC	1	*	*
10.	Colombia	Instituto Nacional de Salud	INS	1	52	https://www.ins.gov.co/
11.	Côte D'Ivoire	Institut National de Santé Publique	INSP	0	*	*
12.	Czech Republic	National Institute of Public Health	SZU	0	*	*
13.	Denmark	Statens Institut for Folkesundhed	SIF	0	*	*
		Statens Serum Institut	SSI	0	*	*
14.	Ethiopia	Ethiopian Public Health Institute	EPHI	1	12	https://ephi.gov.et/
15.	Finland	Finnish Institute for Health and Welfare	FIHW	0	*	*
16.	France	Santé Publique France	SPF	0	*	*
17.	Georgia	Georgia National Center for Disease Control and Public Health	NCDC	0	*	*
18.	Germany	Bundeszentrale für gesundheitliche Aufklärung	BZgA	1	*	*
		Robert Koch Institut	RKI		*	*
19.	Ghana	Noguchi Memorial Institute for Medical Research	NMIMR	0	*	*
		Ghana Health Service	GHS	0	*	*
20.	Guatemala	Centro Nacional de Ciencias de la Salud	CNCS	0	*	*
21.	India	National Centre for Disease Control *(formerly National Institute of Communicable Disease)*	NCDC	0	*	*
22.	Iran Islamic Republic	Institute of Public Health Research	IPHR	0	*	*
23.	Italy	Instituto Superiore di Sanità	ISS	3	*	*
24.	Jordan	Ministry of Health	-	0	15	http://www.moh.gov.jo/
25.	Kazakhstan	National Center for Public Healthcare	NCPH	0	*	*
26.	Kenya	Kenya Medical Research Institute	KEMRI	0	*	*
		Kenya National Public Health Institute	KNPHI	0	*	*
27.	Korea, Rep.	Korea Centers for Disease Control and Prevention/ Korea Disease Control and Prevention Agency	KCDC / KDCA	11	67	http://www.kdca.go.kr/

Count	Country	Name of NPHI (*Based on IANPHI member list)*	Acronym	No. of returns included in the electronic database search	No. of articles included in the search of websites, and social media	NPHI Websites searched
28.	Liberia	National Public Health Institute of Liberia	NPHIL	0	20	https://www.nphil.gov.lr/
29.	Malawi	Public Health Institute Malawi	PHIM	0	*	*
30.	Mexico	Instituto Nacional de Salud Publica	INSP	0	*	*
31.	Mongolia	National Center for Public Health	NCPH	0	*	*
32.	Morocco	Institut Pasteur Du Maroc *(IPM)*	IPM	0	*	*
		National Institute of Hygiene	NIH	0	*	*
		Direction of Epidemiology and Control Diseases, Ministry of Health	-	0	*	*
33.	Mozambique	Instituto Nacional de Saúde	INS	0	*	*
34.	Nigeria	Nigerian Institute of Medical Research	NIMR	4	*	*
1.		Nigeria Centre for Disease Control	NCDC		*	*
1.		National Primary Health Care Development Agency	NPHCDA		*	*
35.	Norway	Norwegian Institute of Public Health	NIPH	2	*	*
36.	Pakistan	Pakistan's National Institute of Health	NIH	0	46	https://www.nih.org.pk/
37.	Panama	Instituto Conmemorativo Gorgas de Estudios de la Salud	ICGES	0	*	*
38.	Russian federation	National Research Center for Preventive Medicine	-	0	*	*
39.	Saudi Arabia	Saudi Centre for Disease Control and Prevention	S. CDC	0	*	*
40.	Sierra Leone	Ministry of Health & Sanitation	-	0	*	*
41.	Somalia	National Institute of Health	NIH	0	*	*
42.	South Africa	National Institute for Communicable Diseases	NICD	2	*	*
43.	Sweden	Public Health Agency of Sweden	-	1	*	*
44.	Thailand	National Institute of Health	NIH	1	*	*
45.	Tunisia	Institut National de la Santé Publique	INSP	0	*	*
46.	Turkey	Refik Saydam National Public Health Agency	-	0	*	*
47.	Uganda	Uganda National Institute of Public Health	UNIPH	0	*	*
1.		Uganda Virus Research Institute	UVRI	0	*	*
48.	Ukraine	Public Health Center *(PHC)* of Ukraine	PHC	0	24	https://www.phc.org.ua/
49.	United Kingdom England	Public Health England	PHE	2	*	*
50.	United states	Centers for Disease Control & Prevention	U.S. CDC	6	*	*
51.	Zambia	Zambia National Public Health Institute	ZNPHI	0	*	*
52.	Tanzania	National Institute for Medical Research	NIMR	1	*	*
Summary statistics				53	277	

*Was not searched among the 8 selected countries
†34 countries with no returns: Afghanistan, Argentina, Bangladesh, Burkina Faso, Cambodia, Cameroon, Côte D'Ivoire, Czech Republic, Denmark, Finland, France, Georgia, Ghana, Guatemala, India, Iran Islamic Republic, Kazakhstan, Kenya, Malawi, Mexico, Mongolia, Morocco, Mozambique, Pakistan, Panama, Russian federation, Saudi Arabia, Sierra Leone, Somalia, Tunisia, Turkey, Uganda, Ukraine, Zambia
‡Articles that describe NPHI activity in more than 1 country are not listed above: 3

Appendix Table 6. Most commonly documented NPHI activities in the COVID-19 response among 18 NPHIs with published documents reflecting their activities

NPHI Function	Roles in the national COVID-19 response
Public health surveillance, problem investigation, and control of risks and threats to public health	- Collecting, analyzing epidemiologic data<br>- Setting case definitions<br>- Screening & testing<br>- Managing laboratory services<br>. Development of guidelines & SOPs<br>. Coordinate the national laboratory network<br>. Producing novel COVID-19 diagnostic technology<br>. Genomic sequencing of the virus<br>. Confirmatory testing<br>. Quality control of diagnostics<br>- Supporting quarantine of positive cases<br>- Contact tracing<br>- Emergency Operations Centers
Public health research	- Creating and maintaining research networks and working groups<br>- Conducting expedited reviews<br>- Conducting or supporting research studies<br>- Bundling data for researchers
Prevention programs and health promotion	- Using websites, social media, and text messaging<br>- Health promotion through multisectoral entities<br>- Working with communities<br>- Establishing hotlines<br>- Health promotion among populations at risk<br>- Supporting vaccination and adverse event reporting
Quality assurance in personal and population-based health services	- Ensuring access to care<br>- Setting up infection prevention and control measures<br>- Ensuring access to Personal Protective Equipment<br>- Producing hospital-based risk assessment tools
Human resources development and training	- Training of laboratorians, contact tracers, vaccination providers, hospital staff<br>- Deploying public health staff to subnational levels<br>- Supporting training platforms and working groups

References

1. de Lusignan S, Lopez Bernal J, Byford R, Amirthalingam G, Ferreira F, Akinyemi O, et al. Influenza and respiratory virus surveillance, vaccine uptake, and effectiveness at a time of cocirculating COVID-19: protocol for the English primary care sentinel system for 2020-2021. JMIR Public Health Surveill. 2021;7:e24341. PubMed https://doi.org/10.2196/24341

2. Del Manso M, Andrianou X, Urdiales AM, Vescio MF, Rota MC, Fabiani M, et al.; Gruppo Referenti regionali della Sorveglianza Integrata COVID-19. La sorveglianza integrata COVID-19 in Italia: output e attività correlate. [COVID-19 integrated surveillance in Italy: outputs and related activities]. Epidemiol Prev. 2020;44(Suppl 2):70–80. PubMed https://doi.org/10.19191/EP20.5-6.S2.105

3. Markus I, Steffen G, Lachmann R, Marquis A, Schneider T, Tomczyk S, et al. COVID-19: cross-border contact tracing in Germany, February to April 2020. Euro Surveill. 2021;26:1–9. PubMed https://doi.org/10.2807/1560-7917.ES.2021.26.10.2001236

4. Petrini C, D'Aprile C, Floridia G, Gainotti S, Riva L, Tamiozzo S. Protection of individual and collective health: ethical-legal issues and opportunities for public health after COVID-19 [cited 2022 May 26.] https://pesquisa.bvsalud.org/global-literature-on-novel-coronavirus-2019-ncov/resource/pt/covidwho-1139101

5. Espitia-Almeida F, Pereira-Lentino R, Quintero-Soto J, Gómez-Camargo D. Covid-19 in Cartagena and the Bolívar Department, Colombia. Current status, perspectives and challenges until the arrival of the vaccine. Heliyon. 2021;7:e06336. PubMed https://doi.org/10.1016/j.heliyon.2021.e06336

6. Abera A, Belay H, Zewude A, Gidey B, Nega D, Dufera B, et al. Establishment of COVID-19 testing laboratory in resource-limited settings: challenges and prospects reported from Ethiopia. Glob Health Action. 2020;13:1841963. **PMID:** 33200686

7. Fretheim A, Brurberg KG, Forland F. Rapid reviews for rapid decision-making during the coronavirus disease (COVID-19) pandemic, Norway, 2020. Euro Surveill. 2020;25:1. PubMed https://doi.org/10.2807/1560-7917.ES.2020.25.19.2000687

8. Oswaldo Cruz Foundation (Fiocruz). Recomendações para o Método Canguru Durante a Pandemia de COVID-19. Portal of Good Practices in Women's, Children's and Adolescents' Health. March 2020. [cited 2022 May 27]. https://portaldeboaspraticas.iff.fiocruz.br/biblioteca/recomendacoes-para-o-metodo-canguru-durante-a-pandemia-de-covid-19/

9. Hong KH, Lee SW, Kim TS, Huh HJ, Lee J, Kim SY, et al. Guidelines for laboratory diagnosis of coronavirus disease 2019 (COVID-19) in Korea. Ann Lab Med. 2020;40:351–60. PubMed https://doi.org/10.3343/alm.2020.40.5.351

10. Hur JY, Kim KW. Crisis learning and flattening the curve: South Korea's rapid and massive diagnosis of the COVID-19 infection. Am Rev Public Adm. 2020;50:606–13. https://doi.org/10.1177/0275074020941733

11. Issac A, Stephen S, Jacob J, Vr V, Radhakrishnan RV, Krishnan N, et al. The pandemic league of COVID-19: Korea versus the United States, with lessons for the entire world. J Prev Med Public Health. 2020;53:228–32. PubMed https://doi.org/10.3961/jpmph.20.166

12. Jeong GH, Lee HJ, Lee J, Lee JY, Lee KH, Han YJ, et al. Effective control of COVID-19 in South Korea: cross-sectional study of epidemiological data. J Med Internet Res. 2020;22:e22103. PubMed https://doi.org/10.2196/22103

13. Kang YJ. Characteristics of the COVID-19 outbreak in Korea from the mass infection perspective. J Prev Med Public Health. 2020;53:168–70. PubMed https://doi.org/10.3961/jpmph.20.072

14. Kim EY, Kim EK, Lee M, Park HK. COVID-19 Public Health Measures During National Assembly Elections of the Republic of Korea. Osong Public Health Res Perspect. 2020;11:158–63. PubMed https://doi.org/10.24171/j.phrp.2020.11.4.03

15. Kim I, Lee J, Lee J, Shin E, Chu C, Lee SK. KCDC risk assessments on the initial phase of the COVID-19 outbreak in Korea. Osong Public Health Res Perspect. 2020;11:67–73. PubMed https://doi.org/10.24171/j.phrp.2020.11.2.02

16. Lee D, Choi B. Policies and innovations to battle Covid-19—a case study of South Korea. Health Policy Technol. 2020;9:587–97. PubMed https://doi.org/10.1016/j.hlpt.2020.08.010

17. Song R, Choi YS, Ko JY. Operating a national hotline in Korea during the COVID-19 pandemic. Osong Public Health Res Perspect. 2020;11:380–2. PubMed https://doi.org/10.24171/j.phrp.2020.11.6.06

18. Rosa MFF, da Silva EN, Pacheco C, Diógenes MVP, Millett C, Gadelha CAG, et al. Direct from the COVID-19 crisis: research and innovation sparks in Brazil. Health Res Policy Syst. 2021;19:10. PubMed https://doi.org/10.1186/s12961-020-00674-x

19. Song R, Kim HS, Yoo SJ, Lee K, Park JH, Jang JH, et al. COVID-19 in nursing facilities: experience in Republic of Korea. Osong Public Health Res Perspect. 2020;11:164–9. PubMed https://doi.org/10.24171/j.phrp.2020.11.4.04

20. de Souza CTV, de Santana CS, Ferreira P, Nunes JA, Teixeira MLB. da Silveira Gouvêa MIF. Caring in the age of COVID-19: lessons from science and society. Cad Saude Publica. 2020;36. https://doi.org/10.1590/0102-311x00115020

21. Andersson S, Aylott N. Nicholas Aylott. Sweden and coronavirus: unexceptional exceptionalism. Soc Sci. 2020;9:232. https://doi.org/10.3390/socsci9120232

22. Onalu CE, Chukwu NE, Okoye UO. COVID-19 response and social work education in Nigeria: matters arising. Soc Work Educ. 2020;39:1037–47. https://doi.org/10.1080/02615479.2020.1825663

23. Apuke OD, Omar B. How do Nigerian newspapers report COVID-19 pandemic? The implication for awareness and prevention. Health Educ Res. 2020;35:471–80. PubMed https://doi.org/10.1093/her/cyaa031

24. Bledsoe M, Captanian A, Somji A. Special report from the CDC: strengthening social connections to prevent suicide and adverse childhood experiences (ACEs): actions and opportunities during the COVID-19 pandemic. J Safety Res. 2021;77:328–33. PubMed https://doi.org/10.1016/j.jsr.2021.03.014

25. Coronado F, Blough S, Bergeron D, Proia K, Sauber-Schatz E, Beltran M, et al. Implementing mitigation strategies in early care and education settings for prevention of SARS-CoV-2 transmission—eight states, September-October 2020. MMWR Morb Mortal Wkly Rep. 2020;69:1868–72. PubMed https://doi.org/10.15585/mmwr.mm6949e3

26. Ding J, Tuan WJ, Temte JL. Managing close contacts of COVID-19 confirmed cases in metropolitan areas in China. J Public Health Manag Pract. 2020;26:345–8. PubMed https://doi.org/10.1097/PHH.0000000000001189

27. Dirlikov E, Fechter-Leggett E, Thorne SL, Worrell CM, Smith-Grant JC, Chang J, et al.; CDC COVID-19 State, Tribal, Local, and Territorial Response Team. CDC deployments to state, tribal, local, and territorial health departments for COVID-19 emergency public health response—United States, January 21–July 25, 2020. MMWR Morb Mortal Wkly Rep. 2020;69:1398–403. PubMed https://doi.org/10.15585/mmwr.mm6939a3

28. Dollard P, Griffin I, Berro A, Cohen NJ, Singler K, Haber Y, et al.; CDC COVID-19 Port of Entry Team. Risk assessment and management of COVID-19 among travelers arriving at designated U.S. airports, January 17–September 13, 2020. MMWR Morb Mortal Wkly Rep. 2020;69:1681–5. PubMed https://doi.org/10.15585/mmwr.mm6945a4

29. Kesselheim AS, Darrow JJ, Kulldorff M, et al. An overview of vaccine development, approval, and regulation, with implications for COVID-19. Health Aff (Millwood). 2021;40:25-32. https://doi.org/10.1377/hlthaff.2020.01620

30. Miralles O, Sanchez-Rodriguez D, Marco E, Annweiler C, Baztan A, Betancor É, et al. Unmet needs, health policies, and actions during the COVID-19 pandemic: a report from six European countries. Eur Geriatr Med. 2021;12:193–204. PubMed https://doi.org/10.1007/s41999-020-00415-x

31. Omaka-Amari LN, Aleke CO, Obande-Ogbuinya NE, Ngwakwe PC, Nwankwo O, Afoke EN. Coronavirus (COVID-19) pandemic in Nigeria: preventive and control challenges within the first two months of outbreak. Afr J Reprod Health. 2020;24(s1):87–97. PubMed

32. Tagliacozzo S, Albrecht F, Ganapati NE. International perspectives on COVID-19 communication ecologies: public health agencies' online communication in Italy, Sweden, and the United States. Am Behav Sci. 2021;65:934–55. https://doi.org/10.1177/0002764221992832

33. Zhang Y, Shi L, Chen H, Wang X, Sun G. Policy disparities in response to the first wave of COVID-19 between China and Germany. Int J Equity Health. 2021;20:86. PubMed https://doi.org/10.1186/s12939-021-01424-3
